# Impact of a double-layer cementing technique on the homogeneity of cementation and the generation of loose bone cement fragments in tibial unicompartmental knee arthroplasty

**DOI:** 10.1186/s12891-019-2929-x

**Published:** 2019-11-13

**Authors:** Christian B. Scheele, Matthias F. Pietschmann, Christian Schröder, Christian Suren, Thomas M. Grupp, Peter E. Müller

**Affiliations:** 1 Department of Orthopaedics, Physical Medicine and Rehabilitation, University Hospital, LMU Munich, Ismaninger Str. 22, 81675 Munich, Germany; 2 Department of Orthopedics and Sports Orthopedics, Technical University Munich, Klinikum rechts der Isar, Ismaninger Str. 22, 81675 Munich, Germany; 3 Aesculap AG Research & Development, Am Aesculap-Platz, 78532 Tuttlingen, Germany

**Keywords:** Cementing technique, UKA, Free bone fragments, Knee arthroplasty, Double-layer

## Abstract

**Background:**

The objective of this study was to evaluate the impact of a single- vs. double-layer cementing technique on morphological cementation and the generation of microscopic cement layers or loose cement fragments in unicompartmental knee arthroplasty (UKA).

**Methods:**

UKAs were implanted in 12 cadaver knees. The specimens were divided into two groups of comparable bone mineral density. Six UKAs were implanted using a single-layer cementing technique (group A) and six UKAs were implanted using a double-layer cementing technique (group B). Morphological cementation was assessed on nine cuts through the implant–cement–bone interface in the frontal plane. Loose bone cement fragments and the microscopically quality of layer formation were evaluated.

**Results:**

Contact between bone and prosthesis was observed in 45.4% of interfaces in group A and 27.8% in group B (*p* = 0.126). The significant increase of areas without visible cement interlocking in the anteroposterior direction in group A (*p* = 0.005) was not evident in group B (*p* = 0.262). Penetration around the peg tended to occur more frequently in group B (67.5% vs. 90.6% *p* = 0.091). Scanning electron microscopy identified no evidence of fissure formations within the bilaminar cement mantle. Free bone cement fragments were documented in 66.7% in both groups with no difference concerning mass (*p* = 1.0).

**Conclusions:**

This in-vitro study showed a tendency towards a more homogenous cementation of tibial UKAs using a double-layer cementing technique, although most of the differences did not reach the level of significance. However, theoretical downsides of the double-layer cementing technique such as an increased formation of free bone fragments or a microscopically fissure formation within the cement layer could not be detected either.

## Background

With adequate patient selection, unicompartmental knee arthroplasty (UKA) has become an effective treatment for anteromedial osteoarthritis of the knee, leading to good long-term survival [1, 2] and better clinical outcomes and patient satisfaction than total knee arthroplasty (TKA) [3–5]. For both TKA and UKA, cementation is considered as standard method of fixation. However, register studies still reveal higher revision rates for UKA than for TKA [6, 7], with aseptic loosening of the tibial component being the most common cause of failure [8]. Aseptic loosing is associated with mechanical fatigue or collapse of the interface between bone, cement and prosthesis. A complete cement mantle with good interdigitation is therefore regarded as prerequisite for good long-term results of UKA [9–12].

The objective of this study was to evaluate the morphological effects of a double-layer cementing technique on the homogeneity of the cement mantle and the interlocking of bone cement with trabecular bone. Moreover, potential negative effects of this technique such as the formation of loose cement particles in the posterior aspect of the joint or a microscopic layer formation within the cement mantle were analyzed.

## Methods

The study received approval from the local ethics committee. Twelve tibial and femoral UKA components (Univation® XF, Aesculap Tuttlingen, Germany) were implanted in the medial compartment of fresh-frozen human cadaver knees using a minimally-invasive approach. In advance, CT-scans (Sensation 64 Somatom, Siemens AG Munich, Germany) of all specimen were obtained to determine bone density and to exclude specimens with osseous irregularities (Sensation 64 Somatom, Siemens Munich, Germany). Based on bone mineral density (BMD), two groups with comparable bone density were formed (Table 1). The Hounsfield units in the anterior, central and posterior regions of the medial tibial plateau were additionally determined for the local determination of bone density [13–15].

**Table 1:** Overview of the 12 specimen, including sex, age, BMD, cementation technique and tibial component size

Specimen	Sex	Age	BMD (mg/mm3)	Cementation technique	Component size
A1	male	78	98	single-layer	T4
A2	male	82	71	single-layer	T4
A3	female	84	81	single-layer	T2
A4	male	53	108	single-layer	T6
A5	male	58	118	single-layer	T4
A6	male	90	143	single-layer	T4
B1	male	54	183	double-layer	T4
B2	male	83	79	double-layer	T6
B3	female	84	76	double-layer	T2
B4	male	53	89	double-layer	T2
B5	male	58	117	double-layer	T4
B6	male	90	135	double-layer	T4

**Fig. 1:**
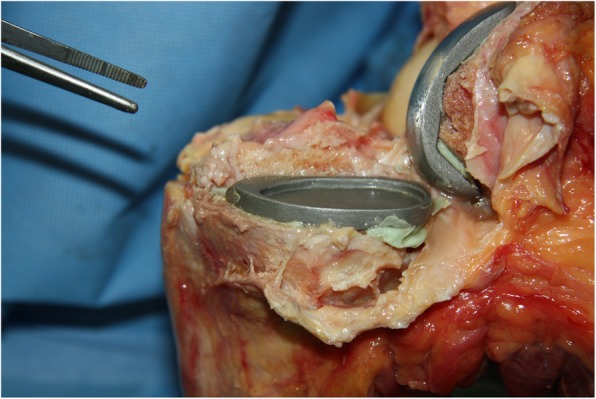
Collection of free fragments of bone cement after complete arthrotomy of the knee joint and after implantation of medial UKA

All implantations were performed by the same experienced surgeon (PEM) following the guidelines of the manufacturer. The appropriate implant size was selected based on preoperative CT measurement and intraoperative findings. After tibial resection, the cancellous bone was conditioned with pulsed lavage (Pulsavac® Plus, Zimmer, Warsaw USA) and saline solution. The tibial and femoral components were cemented in two stages using manually mixed bone cement (Palacos® R 20 g powder/10 ml monomer, Heraeus Medical Wehrheim, Germany). In group A, approximately 10 g of cement were applied manually to the underside of the prosthesis while the resected tibia remained cementless (single-layer cementing technique). In group B, approximately 10 g of cement were applied in equal parts by hand to the underside of the prosthesis plus the tibia. Each tibial component, was carefully placed on the tibia, beginning with the dorsal edge to prevent dorsal cement extrusion, and impacted using a special instrument (Univation® F instruments, Aesculap, Germany). Manual pressure was maintained until the cement cured completely. The joint was then opened and free cement bodies were collected, conserved and quantified using an analytical balance (Sartorius AG, Goettingen, Germany; Fig. 1).

For morphological analysis of the cementation, the tibial prosthesis with the cement phase and the adjacent bone were cut out as blocks. These blocks were then embedded in rectangular aluminium tubes with a transparent resin (Technovit 4004, Heraeus Medical Wehrheim, Germany) and sawed into 10 slices in the coronary plane. To ensure that slices of the different component sizes showed the same implant region, we virtually designed a masterplate for the cutting process (Dassault Systèmes, France) that was manufactured in a rapid-prototyping process (Ultimaker Geldermalsen; Netherlands), attached to the embedded implant and guaranteed that all 10 slices of a specimen had the same thickness.

The cut surfaces were cleaned and tiff-images with a resolution of 100 pixel per mm were acquired of all interfaces (HP Scanjet G3110; Hewlett-Packard Palo Alto, USA). The further evaluation of both sides of the nine serial cuts through the implant–cement–bone interface of the 12 specimens was carried out using Adobe Photoshop CS6 (Adobe, San Jose, USA).

**Fig. 2:**
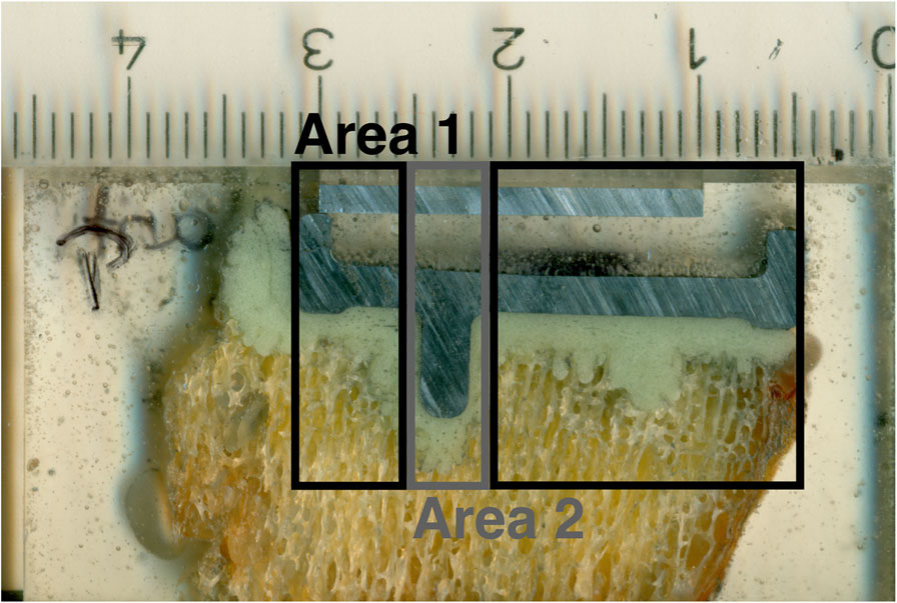
Subdivision of the underside of the prosthesis into area 1 (horizontal underside of the prosthesis; black) and area 2 (area around the anchoring peg; grey)

In order to adequately capture the special geometry of the tibial tray and the associated penetration behavior, the underside was divided into two areas. Area 1 comprised the horizontal part of the prosthesis. Area 2 related to the area around the anchoring peg (Fig. 2).

**Fig. 3:**
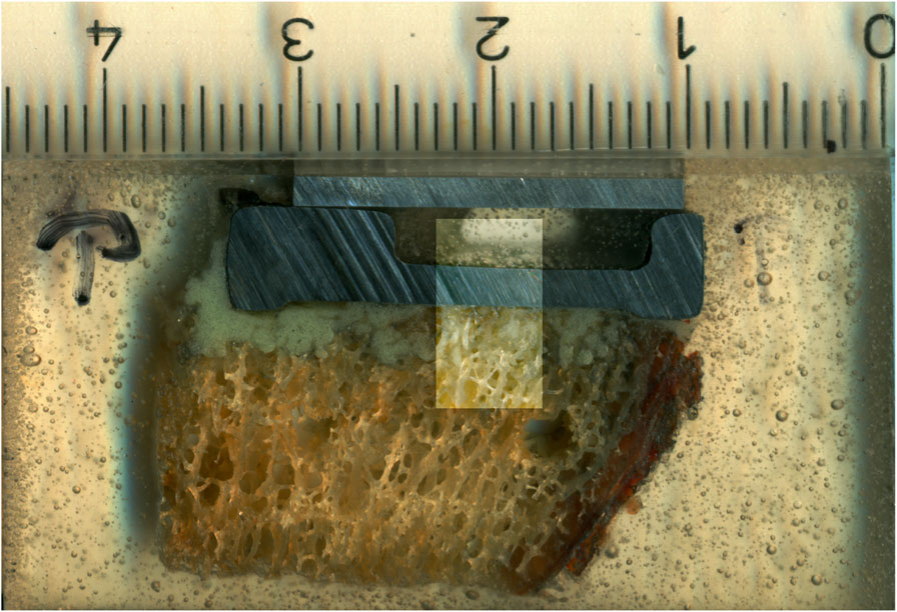
Interruption of the cement mantle with contact between prosthesis and bone

**Fig. 4:**
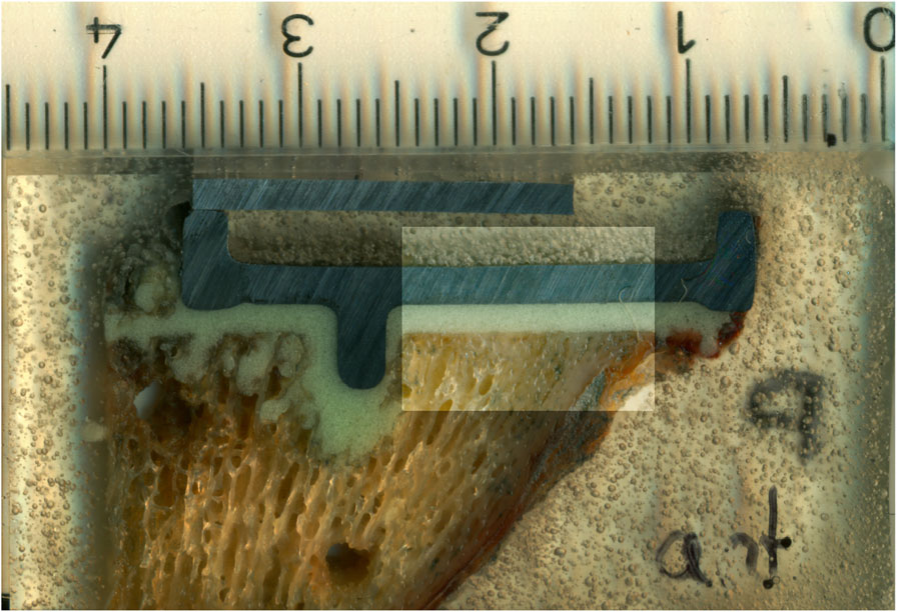
Interruption of the (cement) penetration, i.e. no visible interlocking of the cement with the trabecular bone

**Fig. 5:**
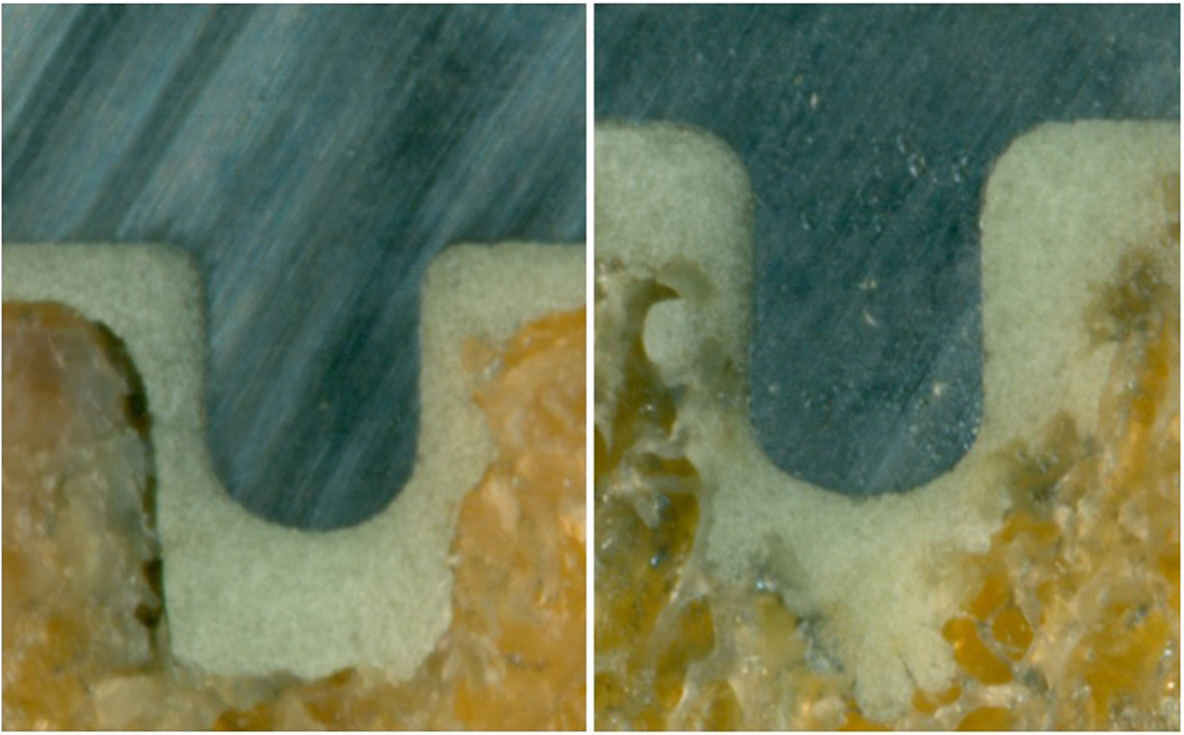
Morphological evaluation of area 2. Left: no penetration; right: visible penetration into trabecular bone

The investigation of area 1 consisted of two indicators. First, the percentage of interfaces with interruptions of the cement mantle, i.e. contact between prosthesis and bone, was determined (Fig. 3). Second, presence and length proportion without cement intrusion into trabecular bone was assessed (Fig. 4). The latter was done by dividing the length without visible cement penetration by the total length of the horizontal bottom side of each tibial tray. Cement intrusion was defined as visible cement penetration into the trabecular bone on the high resolution images. The analysis distinguished between the anterior (cut 9–7), central (cut 6–4) and posterior section (cut 3–1). The investigation in area 2 focused on the proportion of surfaces with visible cement penetration in the area adjacent to the peg (Fig. 5).

In addition to the macroscopic assessment of conventional scans of the cut surfaces, a anterior (front of 8th slice), central (front of 5th slice) and posterior (back of 3rd slice) interface was analyzed by a scanning electron microscope (SEM, Zeiss Evo LS 10, Carl Zeiss Microscopy GmbH, Jena, Germany) and the associated software (SmartSEM V05.04.03.00 Carl Zeiss Microscopy GmbH, Jena, Germany), to approximate a quartering of the specimen. The respective cement-bone-interface was put on the stage of the specimen chamber facing up. A low-vacuum of 1.23 × 10^− 5^ hPa, high voltage of 20.56 kV and magnification of 100 were applied. The surfaces were scanned automatically with an overlap of 15% and the individual tiff-images were manually assembled and analyzed using Adobe Photoshop CS6 (Adobe, San Jose, USA). If fissure formations were apparent, they were defined for the section number, crack number, location of the crack, and length of the crack.

The statistical analysis was performed using GraphPad Prism 5 (GraphPad Software, Inc., La Jolla, CA, USA). A *p*-value < 0.05 was defined to be statistically significant. For statistical evaluation a repeated-measures analysis of variance (ANOVA) was performed to test for significant differences in the morphologic cementation parameters. Prior to analysis, normal distribution was verified (normal P–P plots; *p* < 0.05). Differences of the parameters between the groups were evaluated with a Bonferroni post hoc analysis. Additionally, the correlation between penetration and BMD and the two measurement techniques of bone density was determined using the Spearman’s rank correlation.

## Results

The mean bone density was 154.6 (SD 48.3) HU or 103.3 (SD 26.0) mg/mm^3^ BMD in group A and 168.4 (SD 55.9) HU or 113.3 (SD 41.1) mg/mm^3^ BMD in group B, demonstrating good comparability (*p* = 0.699 [HU]; *p* = 0.937 [BMD]) between the groups. The correlation between HU and BMD was r_s_ = 0.90 (*P* < 0.0001). In group A, the mean HU was 169.8 (SD 73.6) in the anterior, 123.4 (SD 38.9) in the central and 170.5 (SD 53.1) in the posterior section (*p* = 0.203). In group B, the corresponding figures were 185.8 (SD 78.3) HU anterior, 131.0 (SD 49.4) HU central and 188.4 (SD 57.6) HU posterior (*p* = 0.191).

**Fig. 6:**
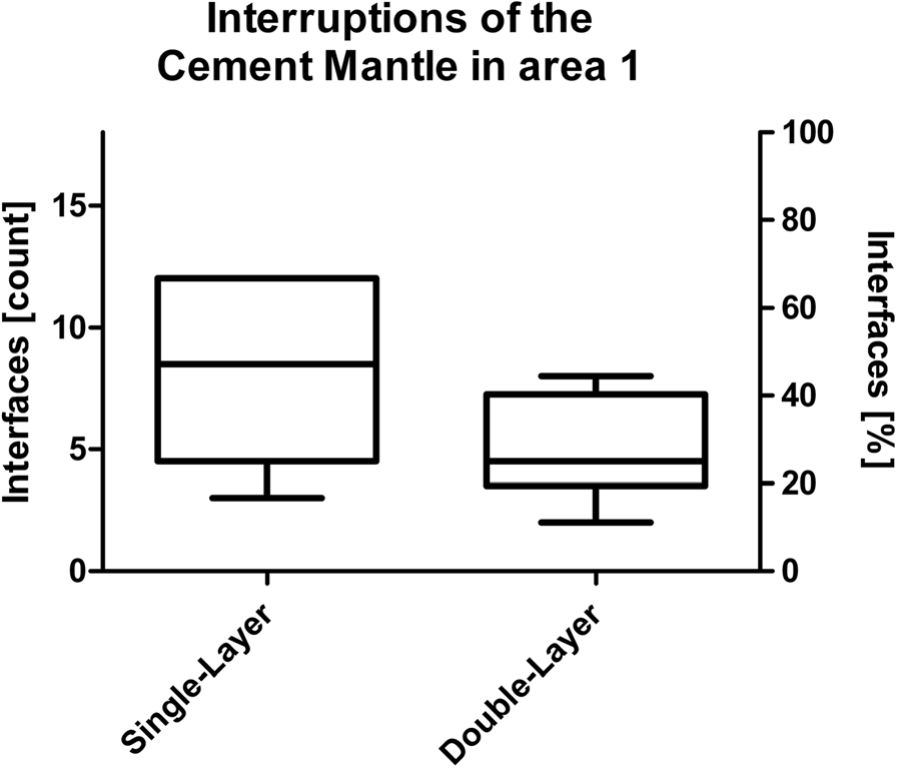
Interruptions of the cement mantle, e.g. interfaces with direct contact between trabecular bone and prosthesis in area 1

In area 1, interruptions of the cement mantle, e.g. direct contact between the bone and the prosthesis were observed in 45.4% of all interfaces in group A and in 27.8% of all interfaces in group B (*p* = 0.126; Fig. 6).

**Fig. 7:**
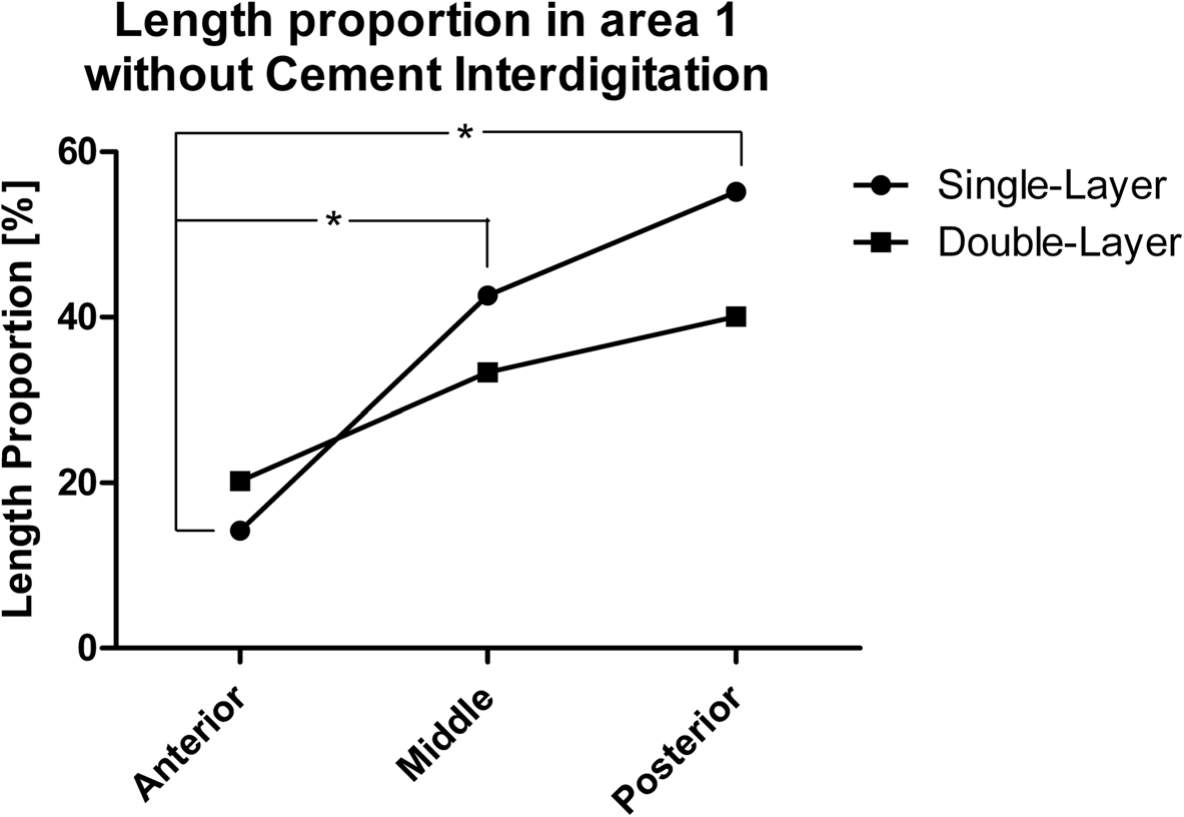
Anteroposterior development of the length portion at the bone-cement-prosthesis interface in area 1 with interruptions of the cement penetration

Areas without visible cement intrusion at cement-bone-interface were observed in 87.8% of all interfaces in group A and 72.2% in group B (*p* = 0.936). The corresponding overall length proportion of area 1 without visible cement intrusion at cement-bone-interface was 37.6% of (SD 10.5%) in group A and 31.0% (SD 21.3%) in group B (*p* = 0.818). However, in the anterior-posterior development, group A showed a proportion of 14.2% (SD 10.5%) of area 1 without visible intrusion in the anterior section, 42.6% (SD 16.7%) in the central section and 55.2% (SD 17.1%) in the posterior section (*p* = 0.005). The corresponding figures for group B were 20.2% (SD 16.8%), 33.3% (SD 23.8%) and 40.1% (SD 28.3%; *p* = 0.262; Fig. 7). The correlation between HU and cement interdigitation was r_s_ = 0.37 (*p* = 0.50) in group A, r_s_ = 0.66 (*p* = 0.18) in group B and r_s_ = 0.50 (*p* = 0.10) with respect to all specimens.

**Fig. 8:**
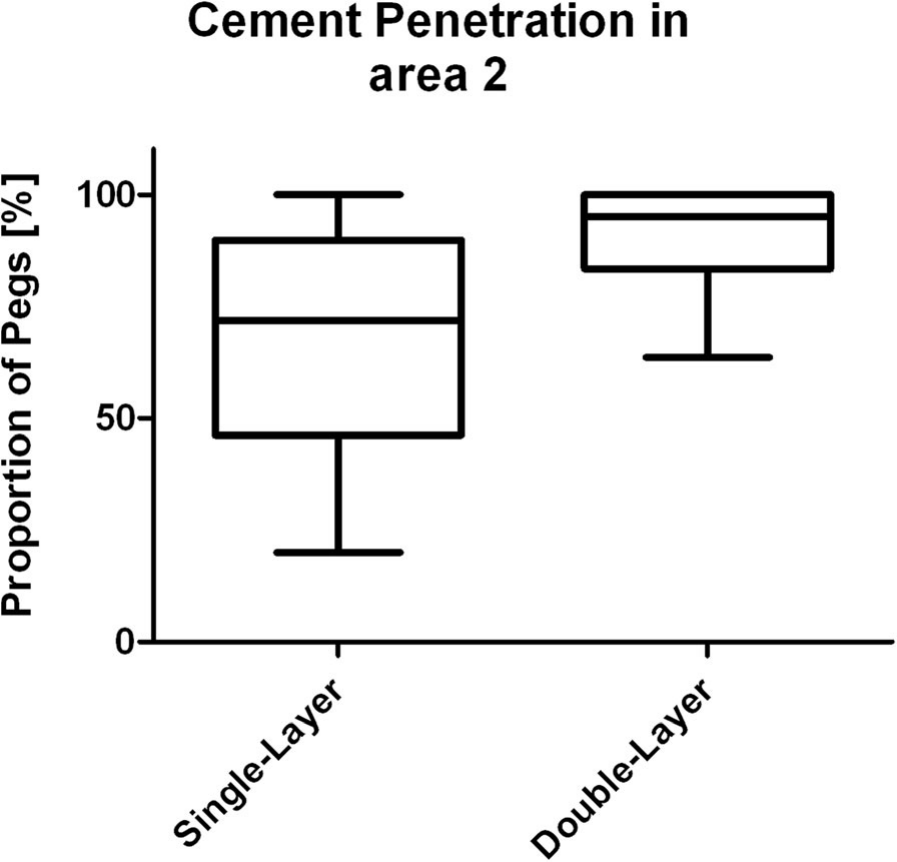
Average proportion of anchoring pegs (area 2) with visible cement penetration

Visible cement penetration in area 2 was less frequently observed in group A with 67.5% than in group B with 90.6% (Fig. 8). However, this difference was not significant (*p* = 0.091).

**Fig. 9:**
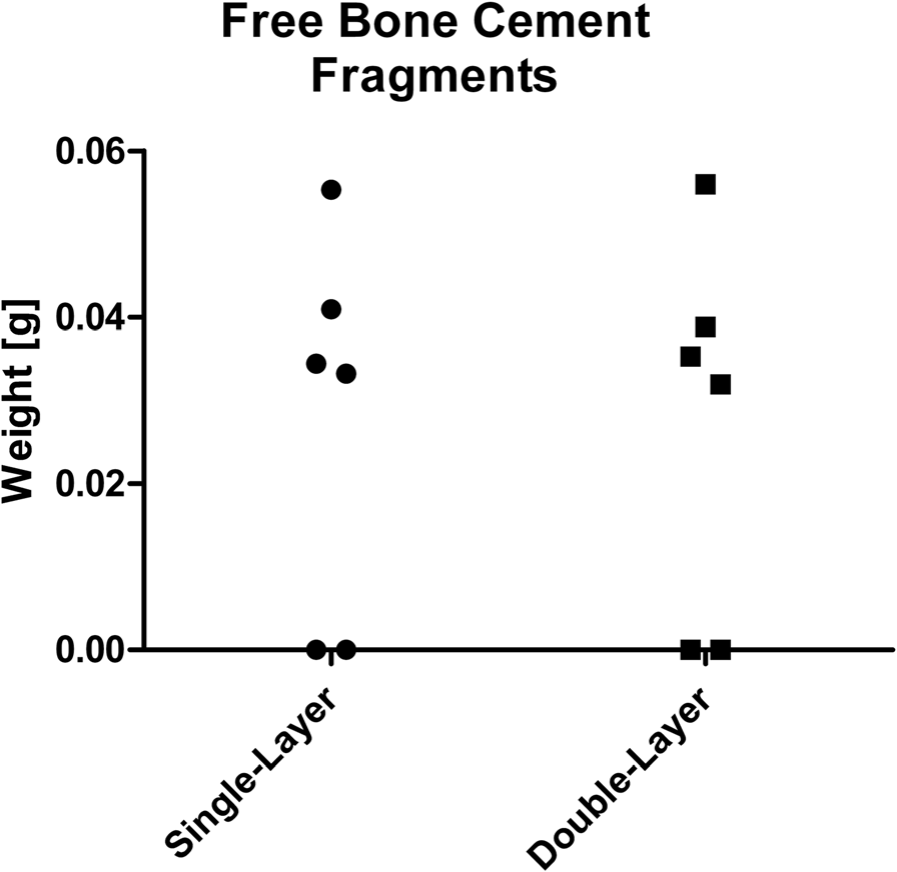
Weight of the free cement bodies

Loose fragments of bone cement could be found in 4 out of 6 cases in both groups (66,7%). Their average weight was 40.1 mg (SD 10.1 mg) in group A and 40.5 mg (SD 10.6 mg) in group B (*p* = 1.0; Fig. 9).

**Fig. 10:**
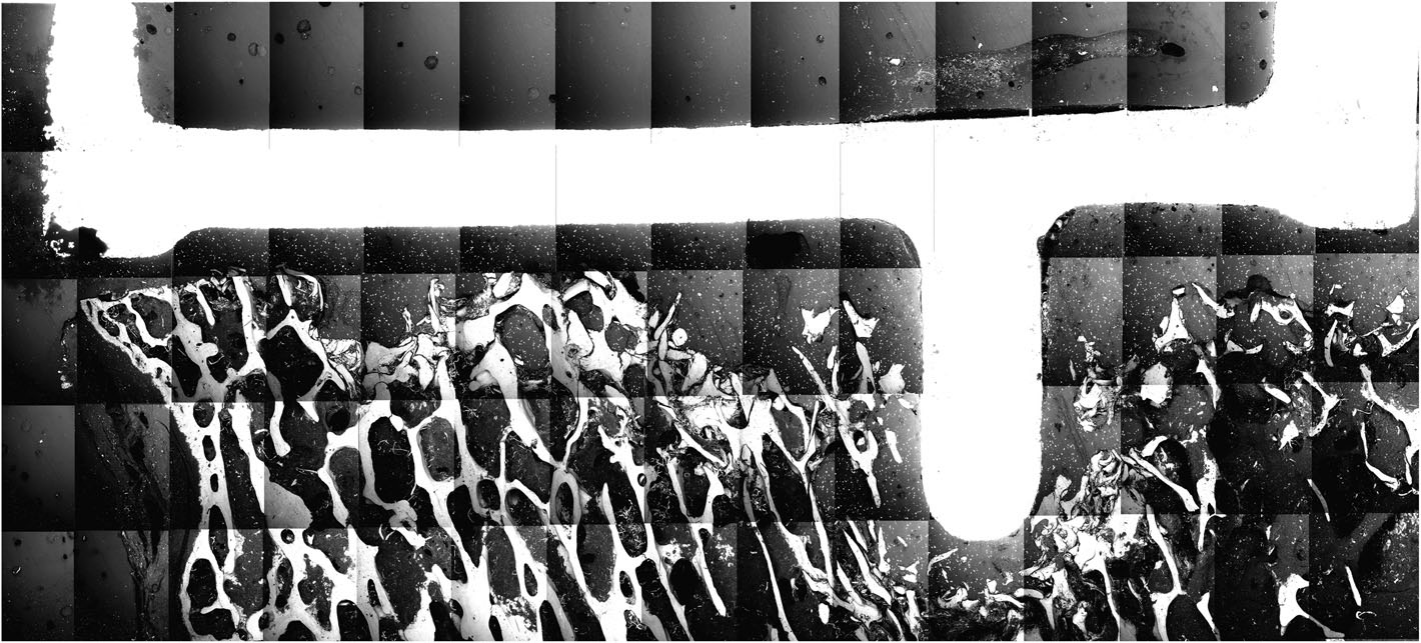
SEM image of a test specimen with double-layer cementation (group B) – overview with no layer formation, cracks or fissure formation apparent

**Fig. 11:**
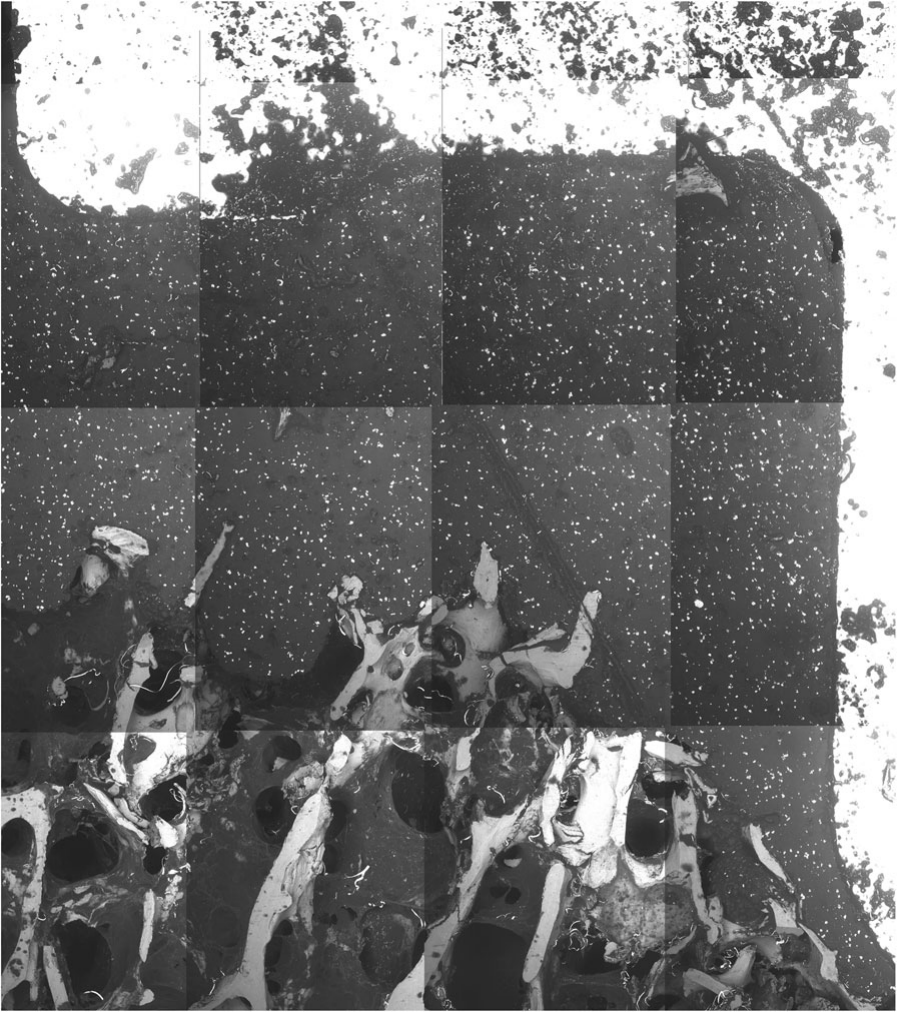
SEM image of a test specimen with double-layer cementation (group B) - magnification; no indication of layer formation, cracks or fissure formation apparent within the cement phase

No evidence of microscopic failure or dissociation within the cement mantle could be detected (Fig. 10; Fig. 11). No cracks were observed in any specimen using both cementing techniques.

## Discussion

Along with other causes such as wear, osteolysis or infection, the main reason for failure in UKA is represented by aseptic loosening of the tibial component [8]. Therefore, a strong interface with cement penetration and mechanical interlocking with trabecular bone is crucial for long-term success [9, 16]. Common clinical practice in cementing UKA is to finger pack bone cement on the prosthesis only. However, former studies using single-layer technique show only limited cement penetration in knee arthroplasty [9, 14, 17]. In particular, a solid fixation in the posterior aspect of the joint seems to be difficult to achieve via a minimally invasive medial approach [13].

With regard to a single- or double-layer cementing technique in knee arthroplasty, only few data is available. Clarius et al. recommended a double-layer technique for femoral UKA in order to ensure a complete filling of drill holes [18]. Furthermore, Vanlommel et al. reported a significantly higher cement penetration depth using a double-layer cementing technique in a study on sawbones [19]. However, according to Hauptmann et al., a thicker cement layer is associated with excess cement bodies [20]. Grupp et al. showed no overall difference in cement penetration depth after implanting tibial UKA in an in vitro cadaver study [21].

This study assesses potential morphologic advantages and disadvantages of a bilaminar cementing technique in UKA. Therefore, the first goal of this study was to assess, whether a double-layer cementation technique improves the homogeneity of the cement mantle and its penetration into trabecular bone. The second goal was to quantify theoretical concerns of the double-layer application of bone cement, like failure at the interface between the two layers of cement and the generation of loose cement fragments in the posterior aspect in the joint.

Concerning the macroscopic examination, there was an overall trend in favor of the double-layer cementing technique, but no significant differences. The proportion of cement-bone-prosthesis interfaces with direct contact between the prosthesis and trabecular bone was 45.6% in group A („single layer“) and 27.8% in group B („double layer“) in area 1 (*p* = 0.126). Cement penetration into trabecular bone in area 2 was visible on 67.5% of all interfaces in group A and 90.6% in group B (*p* = 0.096). The significant increase of the length proportion of area 1 without cement interdigitation in the anteroposterior direction in group A (*p* < 0.005) was not evident in group B (*p* = 0.262). The latter might be important, as the posterior part of the joint is often regarded as a weak point of cemented fixation [18, 22], especially using a minimally-invasive approach [13]. One reason is the difficulty to reach the posterior aspect of the joint with a conventional jet lavage [14, 22, 23]. Another might be the fact that the component is inserted parallel to the bone surface, which makes orthogonal pressure application on the bone cement challenging, in particular when using a minimally invasive approach [13].

Although there were no significant advantages of double-layer cementation in the macroscopic examination of the interfaces, the concern about potential disadvantages could not be confirmed either. In our study, no microscopic differences between both techniques could be detected. In particular, neither morphological layers, nor the generation of cracks or fissures were recognized using SEM with a low-vacuum and a BSE-detector. It was not possible to identify the cement-on-cement interface. These findings are consistent with earlier observations from cement-within-cement technique in revision hip arthroplasty [24–26]. This technique involves the retention of the existing bone-cement-interface and the recementing of the stem within the completely or partially maintained old cement mantle. Ishihara et al. defined cement failure as the coalescence of several cracks, where cracks occur between voids within the cement [27]. According to Weinrauch et al., the absence of a visible layer formation is probably due to a diffusion-based molecular interdigitation and fusion of the two applied cement phases [25]. Wilson et al. investigated the biomechanical properties of a bilaminar cementation on Sawbones under cyclic loading. They could neither detect a macroscopic failure of the bilaminar cementation nor identify a boundary between the cement layers using SEM [28]. Furthermore, the cement layers in the double-layer cementing technique are not hardened during the implantation of the tibial tray and the remaining MMA monomer ensures a chemical bonding without any layer formation after curing. Microscopic inspection of the cement mantle using SEM can be regarded as an established method and was applied by other authors before [10, 26, 29, 30].

Moreover, there was no indication of an increase in the mass of free cement fragments in the posterior aspect of the joint (*p* = 1.0). Their frequent occurrence in four out of six specimen in both groups is in accordance with a study by Hauptmann et al., according to which free cement fragments after minimally-invasive implantation of UKA represent an underestimated problem [20]. Elmadag et al. documented the radiologic occurrence of posteromedial cement extrusion after UKA in 18,6% of cases [31]. Bhuta et al. reported a case of tibial nerve impingement as a consequence of posterior cement extrusion after a minimally-invasive UKA [32]. Our results are in line with this case and other observation of extruded cement remnants in UKA [28, 33, 34].

In summary, the significant advantage of two-layer cementation on cement penetration described in Sawbones could not be reproduced under minimally-invasive surgical conditions in human tibia. However, no indications of adverse effects of two-layer cementation were observed. These results are consistent with previous results, that described no differences between the single and double-layer cementing technique of unicompartmental tibial components with regard to failure load [21].

This study is subject to potential limitations. First, the present in-vitro examination on cadaver legs did not simulate blood circulation. It was postulated from hip arthroplasty experiments that blood flow might have a negative influence on cement penetration [35, 36]. For example, Greenwald et al. postulated a 37% decrease in shear strength due to blood inclusion [37]. Li et al. [24] quantified the negative influence of a blood-bone marrow layer on tensile strength and shear force at 80 and 85%, respectively, and described a microscopically visible layer of less than 100 μm between the cement phases. However, this view is controversial as MacDonalds et al. did not find any significant traces of blood at the interface between bone and cement or blood inclusions within the cement in an in-vivo animal model [12]. In addition, Rudol et al. stated that only large amounts of highly viscous liquids (e.g. bone marrow) weaken the interface. In contrast, low-viscosity fluids like blood did not significantly influence the interface strength [38]. Moreover, bleeding can be prevented during implantation by the application of a tourniquet. Second, common manual pressure application during curing of the cement was applied in order to achieve the best possible simulation of surgical conditions. Although one experienced surgeon performed all implantations, an absolute consistence of force can therefore not be secured. Third, human specimen were not randomly distributed to the groups due to the rather small sample size. To ensure comparable conditions, their allocation was performed as matched-pairs of comparable bone mineral density.

## Conclusions

The present study does not provide any indications of negative effects caused by a double-layer cementing technique such as an increase in the formation of free bone fragments or a microscopically layer formation in tibial UKA. However, improvements in the morphologic homogeneity of the cement mantle in area 1 or the improved average penetration in area 2 do not reach the level of significance. Therefore, the effectiveness of a double-layer cementation technique should be investigated further in randomized controlled clinical trials.

## Data Availability

The datasets used and/or analysed during the current study are available from the corresponding author on reasonable request.

## Abbreviations

BMD: Bone mineral density
BSE: Back-scatter detector
CT: Computed tomography
et al.: et alii
Fig.: Fig.
HU: Hounsfield units
ROI: Region of interest
SD: Standard deviation
SEM: Scanning electron microscope
TKA: Total knee arthroplasty
UKA: Unicompartmental knee arthroplasty
